# MAPKs/AP-1, not NF-κB, is responsible for MCP-1 production in TNF-α-activated adipocytes

**DOI:** 10.1080/21623945.2022.2107786

**Published:** 2022-08-08

**Authors:** Xiaoyu Zhang, Zhuangzhuang Liu, Wenjing Li, Yuan Kang, Zhenlu Xu, Ximeng Li, Yuan Gao, Yun Qi

**Affiliations:** Institute of Medicinal Plant Development, Chinese Academy of Medical Sciences & Peking Union Medical College, Beijing, Haidian, China

**Keywords:** Obesity, adipocyte, MCP-1, MAPKs/AP-1, NF-κB

## Abstract

Obesity is associated with the infiltration of monocytes/macrophages into adipose tissue in which MCP-1 plays a crucial role. But the regulatory mechanism of MCP-1 expression in adipocytes is not well defined. Our results demonstrated that TNF-α induced abundant MCP-1 production in adipocytes, including 3T3-L1 pre- (≈ 9 to 18-fold), mature adipocytes (≈ 4 to 6-fold), and primary adipocytes(< 2-fold), among which 3T3-L1 pre-adipocytes showed the best reactiveness. Thus, 3T3-L1 pre-adipocytes were used for the most of following experiments. At the transcriptional level, TNF-α (20 ng/mL) also promoted the mRNA expression of MCP-1. It is well recognized that the engagement of TNF-α with its receptor can trigger both NF-κB and AP-1 signalling, which was also confirmed in our study (5-fold and 2-fold). Unexpectedly and counterintuitively, multiple NF-κB inhibitors with different mechanisms failed to suppress TNF-α-induced MCP-1 production, but rather the inhibitors for any one of MAPKs (JNK, ERK and p38) could do. This study, for the first time, reveals that MAPKs/AP-1 but not NF-κB signalling is responsible for MCP-1 production in TNF-α-activated adipocytes. These findings provide important insight into the role of AP-1 signalling in adipose tissue, and may lead to the development of therapeutical repositioning strategies in metaflammation.

**Abbreviations:** AP-1, activator protein-1; CHX, cycloheximide; IR, insulin resistance; MAPK, mitogen-activated protein kinase; NF-κB, nuclear factor κB; RT-qPCR, quantitative real-time PCR; T2DM, type 2 diabetes mellitus; TRE, triphorbol acetate-response element.

## Introduction

Obesity is characterized as a chronic state of low-grade inflammation (termed metaflammation) with progressive immune cell infiltration into adipose tissue. Over the past decades, the worldwide epidemic has become a major health concern, because it contributes to high mortality due to an increasing incidence of metabolic disorders, including insulin resistance (IR), type 2 diabetes mellitus (T2DM), atherosclerosis, liver diseases, and some cancers [1]. Although the underlying mechanisms for the link between obesity and these diseases are not fully understood, it is clear that adipose tissue as a special microenvironment and immune cells as major players, are both essential elements for initiating and sustaining metaflammation [2–4].

In the early 1990s, it was first identified that obesity was associated with increased TNF-α expression in adipose tissue of obese mice [5]. Moreover, TNF-α even can autoregulate positively its own biosynthesis in the adipose tissue, thus contributing to the maintenance of high TNF-α level in obesity [6]. Through its receptor (TNF-R1) on adipocytes, TNF-α is able to activate nuclear factor-kappa B (NF-κB) and MAPKs/activator protein-1 (AP-1) signalling and ultimately leads to the production of pro-inflammatory mediators [7–9]. Although moderate inflammation is essential for healthy adipose tissue expansion and remodelling [10], excessive adipocyte inflammation may contribute to a serial of metabolic disorders and other obesity comorbidities.

MCP-1 is a crucial pro-inflammatory chemokine for adiposity. Unlike some other adipose tissue-produced cytokines (e.g. TNF-α, IL-6, and IL-1) which mainly originate in non-fat cells [11], the basal release of MCP-1 primarily occurred in pre-adipocytes (*i.e*. adipocyte progenitor cells) [12,13] or mature adipocytes [14,15]. In fact, MCP-1 may have a more profound effect on obesity-associated risks than other adipokines [16]. Especially in the initiation stage of adipose tissue inflammation, adipocytes rather than macrophages release abundant MCP-1 to recruit monocytes into adipose tissue [17,18]. Subsequently these monocytes are polarized into pro-inflammatory macrophages to secrete inflammatory cytokines including TNF-α, which in turn stimulates adipocytes to produce more MCP-1 [12,16]. This interaction between adipocytes and macrophages perpetuates a vicious inflammatory loop, thereby leading to the immortal inflammatory state around the adipose tissue [19,20].

In the resting cells, NF-κB is sequestered in the cytoplasm as the inactive form. Upon TNF-α stimulation, p50/p65 heterodimer (the predominant NF-κB isoform) translocates to nucleus to initiate the transcription of various cytokines including MCP-1. Nevertheless, we accidentally found that inactivation of NF-κB did not affect TNF-α-induced MCP-1 production in either pre- or mature adipocytes. Similar situations that NF-κB is dispensable for MCP-1 production have also been found in other cells [21–23]. Given the fact that MAPKs/AP-1, another signalling responsible for MCP-1 production, can also be triggered by TNF-α, we hypothesized that MAPKs/AP-1 should be the dominant controller. Accordingly, the present study aimed to investigate the contributions of NF-κB and MAPKs/AP-1 to MCP-1 production in TNF-α-activated adipocytes by using their respective inhibitors.

## Results

### TNF-α causes MCP-1 production in adipocytes

**Figure 1.**
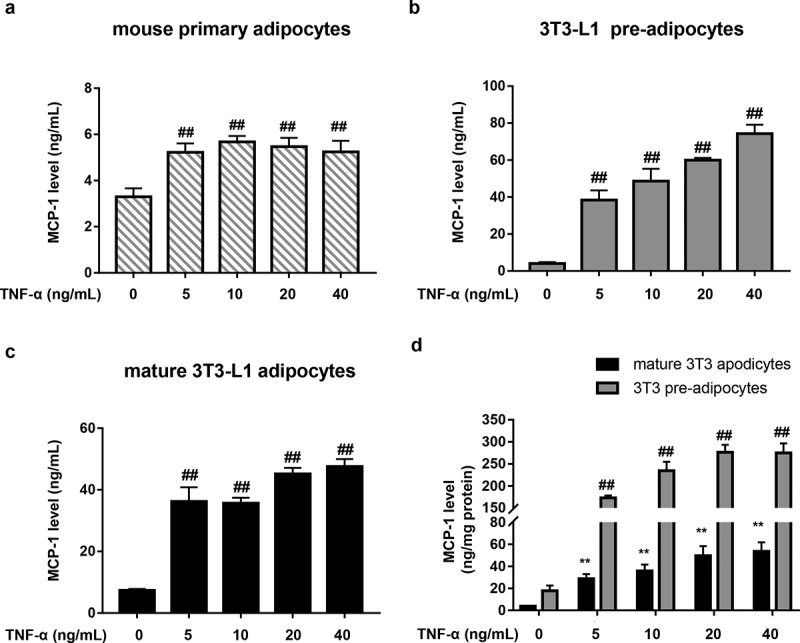
TNF-α causes MCP-1 production in adipocytes. (a – c) Effects of TNF-α on MCP-1 production in mouse primary adipocytes (a), 3T3-L1 pre-adipocytes (b) and 3T3-L1 mature adipocytes (c). The cells were stimulated with TNF-α at different concentrations (0–40 ng/mL) for 24 h. Subnatant or supernatant MCP-1 was determined by ELISA. All data were presented as mean ± SD (n = 3). ^##^*P* < 0.01 *versus* normal control group. (d) Supernatant MCP-1 level (ng/mg protein) of 3T3-L1 mature adipocytes and pre-adipocytes after standardizing protein. All data were presented as mean ± SD (n = 3). ^##^*P* < 0.01 *versus* normal control group in 3T3-L1 pre-adipocytes; **P < 0.01 *versus* normal control group in 3T3-L1 mature adipocytes.

MCP-1 is one of key chemokines that initiate obesity-induced inflammation and monocyte chemoattractant activities [24]. In agreement with previous findings from other researchers [25,26], we also found that resting adipocytes could release a certain amount of MCP-1 (Figure 1) which may recruit monocytes to adipose tissue where they differentiate into macrophages [17]. TNF-α released from these macrophages can further increase MCP-1 production of adipocytes [27]. Indeed, our data showed that TNF-α (5–40 ng/mL) significantly elevated MCP-1 in the culture medium of adipocytes, including mouse primary adipocytes, 3T3-L1 pre- and mature adipocytes (Figure 1(a – c)). However, the reactivity of primary adipocytes (< 2-fold) in response to TNF-α was obviously weaker than that of 3T3-L1 pre-adipocytes (≈ 9 to 18-fold) and 3T3-L1 mature adipocytes (≈ 4 to 6-fold) (Figure 1(a – c)). In the case of equal intracellular protein, 3T3-L1 pre-adipocytes still exhibited higher reactivity to TNF-α in contrast to 3T3-L1 mature adipocytes (Figure 1(d)). Based on the above results, 3T3-L1 pre-adipocytes and 20 ng/mL TNF-α were used for the most of subsequent experiments.

### TNF-α increases MCP-1 mRNA level in 3T3-L1 pre-adipocytes

**Figure 2.**
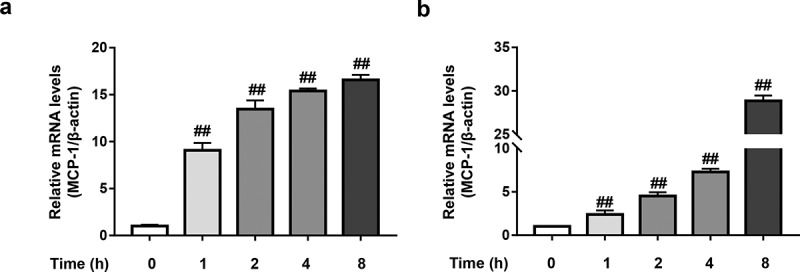
TNF-α increases MCP-1 mRNA level in 3T3-L1 pre-adipocytes. (a) Effect of TNF-α on MCP-1 mRNA level in the absence of CHX in 3T3-L1 pre-adipocytes. 3T3-L1 pre-adipocytes were stimulated by TNF-α (20 ng/mL) for indicated time (0–8 h). Total RNA was extracted and reversely transcribed and the mRNA level was determined by RT-qPCR assays. (b) Effect of TNF-α on MCP-1 mRNA level in the presence of CHX in 3T3-L1 pre-adipocytes. 3T3-L1 pre-adipocytes were pretreated with CHX (500 nM). Two hours later, 3T3-L1 pre-adipocytes were stimulated by TNF-α (20 ng/mL) for indicated time (0–8 h). Total RNA was extracted and reversely transcribed and the mRNA level was determined by RT-qPCR assays. All data were presented as mean ± SD (n = 3). ^##^*P* < 0.01 *versus* normal control group.

We next investigated the effect of TNF-α on MCP-1 mRNA level in 3T3-L1 pre-adipocytes by RT-qPCR assays. As shown in Figure 2(a), TNF-α (20 ng/mL) was able to significantly elevate the mRNA level of MCP-1 which reached 9.1-fold of the base level at 1 h and gradually increased to 16.6-fold at 8 h. To avoid the interference of other mediators induced by TNF-α, the protein synthesis inhibitor cycloheximide (CHX) was used. The obtained data showed that in the presence of CHX, MCP-1 mRNA level still increased with time after TNF-α stimulation (Figure 2(b)), indicating that TNF-α directly increased MCP-1 mRNA level in 3T3-L1 pre-adipocytes.

### TNF-α activates NF-κB and AP-1 pathways in 3T3-L1 pre-adipocytes

**Figure 3.**
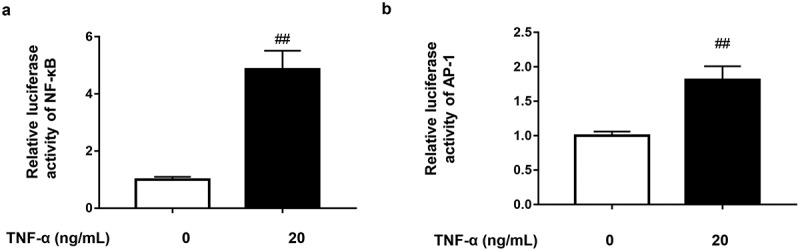
TNF-α activates NF-κB and AP-1 pathways in 3T3-L1 pre-adipocytes. (a) Effect of TNF-α on NF-κB signal in 3T3-L1 pre-adipocytes. The cells transfected with pNFκB-TA-luc plasmid were stimulated by TNF-α (20 ng/mL) for 4 h. The luciferase activity of cell lysate was measured using the luciferase assays. (b) Effect of TNF-α on AP-1 signal in 3T3-L1 pre-adipocytes. The cells transfected with pAP-1-TA-luc plasmid were stimulated by TNF-α (20 ng/mL) for 4 h. The luciferase activity of cell lysate was measured using the luciferase assays. All data were presented as mean ± SD (n = 3).   ^##^*P* < 0.01 *versus* normal control group.

To our knowledge, the engagement of TNF-α with TNF-R1 activates two major transcription factors NF-κB and AP-1 which are responsible for the transcriptional regulation of MCP-1 [9,28]. Therefore, we next investigated the effect of TNF-α on the activities of these two transcriptional factors by using the luciferase reporter gene system. As expectedly, TNF-α (20 ng/mL) indeed could activate both NF-κB and AP-1 in 3T3-L1 pre-adipocytes, which respectively showed about 5-fold and 2-fold fluorescence intensity increase in contrast to normal control (Figure 3).

### MCP-1 production is independent of NF-κB activation in TNF-α-activated 3T3-L1 adipocytes

**Figure 4.**
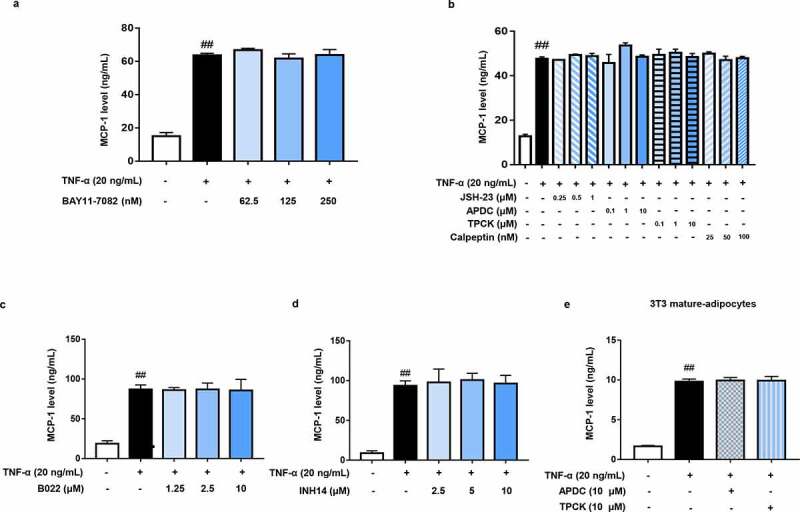
MCP-1 production is independent of NF-κB activation in TNF-α-activated adipocytes. (a – d) Effects of BAY11-7082, JSH-23, APDC, TPCK, calpeptin, B022, and INH14 on MCP-1 production in TNF-α-activated 3T3-L1 pre-adipocytes. Cells were pretreated with multiple NF-κB inhibitors for 1 h and followed by TNF-α (20 ng/mL) stimulation for 24 h. Supernatant MCP-1 production was determined by ELISA. (e) Effects of APDC and TPCK on MCP-1 production in TNF-α-activated 3T3-L1 mature adipocytes. 3T3-L1 mature adipocytes were pretreated with APDC and TPCK for 1 h and followed by TNF-α (20 ng/mL) stimulation for 24 h. Supernatant MCP-1 production was determined by ELISA. All data were presented as mean ± SD (n = 3). ^##^*P* < 0.01 *versus* normal control group.

Previous studies reported there existed κB site in the MCP-1 promoter region [27]. And NF-κB signalling is important for the cytokine-stimulated MCP-1 production in astrocytes and renal cells [29,30]. In theory, MCP-1 transcription should be regulated by NF-κB. Therefore, we evaluated the contribution of NF-κB activation to MCP-1 production in TNF-α-stimulated 3T3-L1 adipocytes. To inhibit the activation of NF-κB, an IκBα phosphorylation inhibitor BAY11-7082 was first used [31]. Surprisingly and counterintuitively, it failed to affect TNF-α-induced MCP-1 production (Figure 4(a)). To rule out the off-target effect, we further selected other inhibitors which can inhibit NF-κB signalling pathway via various targets, including JSH-23 (an inhibitor of p65 nuclear translocation) [32], APDC and TPCK (the NF-κB transcription inhibitors) [33,34] and calpeptin (an inhibitor of IκBα degradation) [35]. Consistently, all of these NF-κB inhibitors didn’t affect TNF-α-induced MCP-1 production in 3T3-L1 pre-adipocytes (Figure 4(b)).

NF-κB activation is not only induced by IKKβ (classical NF-κB pathway), but also regulated by alternative NF-κB pathway which involves NIK-mediated IKKα activation. In order to clarify the influence of alternative NF-κB pathway on MCP-1 production, the selective NIK inhibitor B022 was used [36]. Analogously, B022 could not affect MCP-1 production in TNF-α-stimulated 3T3-L1 pre-adipocytes (Figure 4(c)). To further confirm the above results, we deliberately chose INH14, a dual inhibitor of IKKα/β [37]. As expected, MCP-1 production was not affected when both classical and alternative NF-κB signalling pathways were blocked (Figure 4(d)). Consistent result was also obtained in mature adipocytes (Figure 4(e)). These results clearly demonstrate that TNF-α-induced MCP-1 production is independent of NF-κB activation in adipocytes.

### MAPKs is responsible for MCP-1 production in TNF-α-activated 3T3-L1 adipocytes

**Figure 5.**
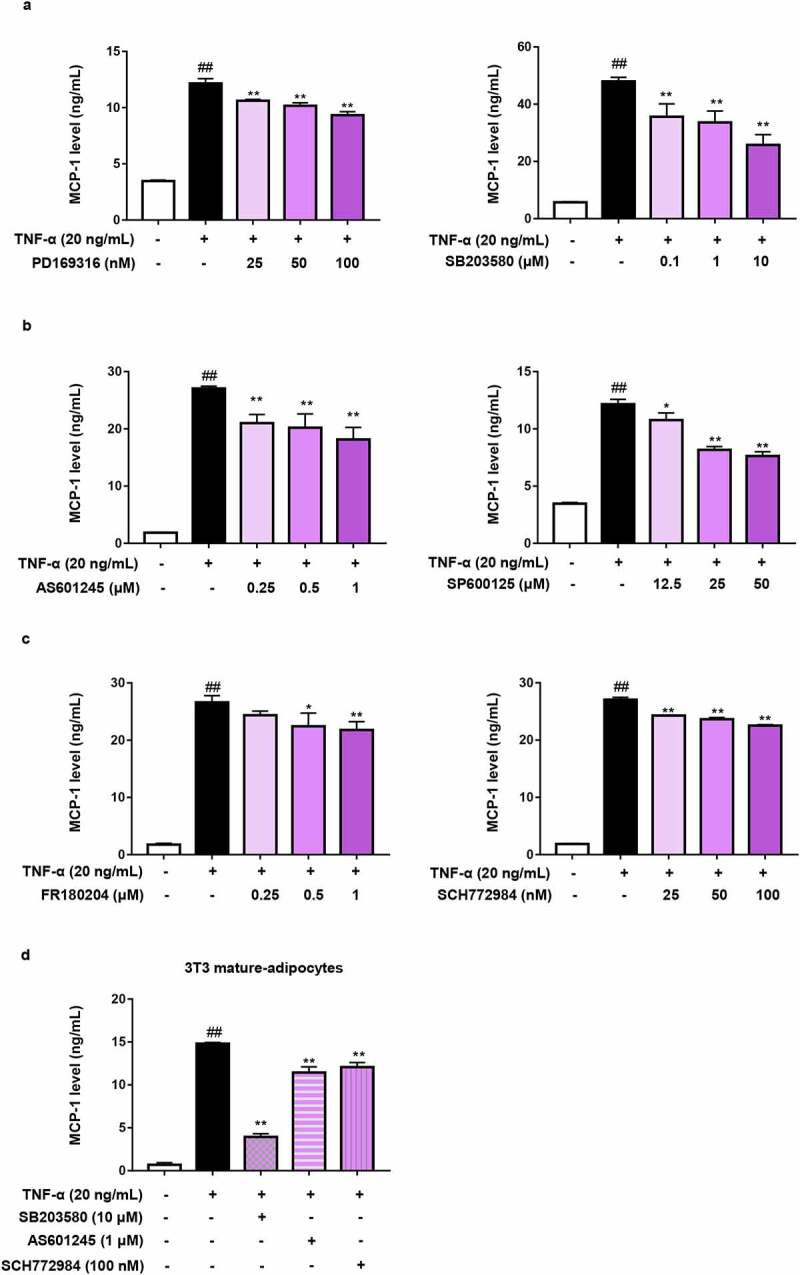
MAPKs is responsible for MCP-1 production in TNF-α-activated adipocytes. (a – c) Effects of inhibitors for (a) p38, (b) JNK, (c) ERK on TNF-α-induced MCP-1 production in 3T3-L1 pre-adipocytes. Cells were pretreated respectively with p38, JNK and ERK inhibitors for 1 h and followed by TNF-α (20 ng/mL) stimulation for 24 h. Supernatant MCP-1 production was determined by ELISA. (d) Effects of the inhibitors for p38, JNK, ERK on TNF-α-induced MCP-1 production in 3T3-L1 mature adipocytes. 3T3-L1 mature adipocytes were pretreated with SB203580, AS601245 and SCH772984 for 1 h and followed by TNF-α (20 ng/mL) stimulation for 24 h. Supernatant MCP-1 production was determined by ELISA. All data were presented as mean ± SD (n = 3). ^##^*P*< 0.01 *versus* normal control group. *P < 0.05 and **P < 0.01 *versus* TNF-α alone group.

Besides NF-κB, AP-1 is another transcription factor that binds to the promoter region of MCP-1 gene and regulates MCP-1 expression. Therefore, we investigated the role of AP-1 activation in TNF-α-mediated MCP-1 production. As we know, AP-1 is regulated by MAPKs family, including JNK, ERK and p38 MAPK. To inhibit the activation of AP-1 signalling, p38 inhibitors (PD169316 and SB203580), JNK inhibitors (SP600125 and AS601245) and ERK inhibitors (FR180204 and SCH772984) were used [38–43]. As shown in Figure 5(a – c), inhibiting anyone of three MAPKs could significantly decrease TNF-α-induced MCP-1 production in 3T3-L1 pre-adipocytes. Similar result was also obtained in mature adipocytes (Figure 5(d)), indicating that MAPKs is responsible for MCP-1 production in TNF-α-activated adipocytes.

## Discussion

Low-grade inflammation in adipose tissue (especially visceral adipose tissue) is recognized as an important contributor to obesity-induced metabolic disorders [44]. The adipose tissue contains mature adipocytes and the stromal vascular fraction mainly including macrophages and adipocyte progenitor cells (*i.e*. pre-adipocytes) [45]. Although pro-inflammatory macrophages play the critical roles in maintaining the chronic inflammatory state and giving rise to metabolic dysfunctions [46], adipocytes (mature adipocytes and pre-adipocytes) are also actively involved in metaflammation process. Especially in the early stage of obesity, pre-adipocytes, the precursor cells of mature adipocytes, release abundant MCP-1 to initiate inflammation [12,13,18].

3T3-L1 adipocyte model serves as an excellent *in vitro* model that contributes to understanding of adipocyte biology and dysfunction [47]. As the first discovered pro-inflammatory cytokine released by adipose tissue [5], TNF-α is the main driver for inducing lipid dysregulation and inflammation in adipocytes, such as stimulating lipolysis, reducing lipid accumulation, decreasing adiponectin secretion, and increasing the secretion of pro-inflammatory adipokines and cytokines (e.g. MCP-1, IL-6 and IL-1β) [48]. In the present study, we focused on TNF-α-induced production of MCP-1, a crucial pro-inflammatory chemokine for adiposity. As we know, TNF-α can bind to its receptor TNF-R1, which highly expresses on adipocytes, to activate two main signal transduction pathways, namely NF-κB and AP-1 [9]. Actually, NF-κB binding sites (A1 and A2) were identified in the promoter region of MCP-1/JE gene [49]. Generally, the transcription factor NF-κB binds to its *cis*-acting elements to initiate transcription of MCP-1. Unexpectedly, our results showed that blocking the classical or alternative NF-κB signalling barely affected MCP-1 production (Figure 3(a) and Figure 4) although TNF-α indeed could activate NF-κB signalling, suggesting that NF-κB activation is dispensable for MCP-1 production in TNF-α-stimulated adipocytes.

In addition to NF-κB binding sites, the 5’-flanking region of MCP-1/JE gene contains another *cis*-acting transcription regulatory element, triphorbol acetate-response element (TRE), which is recognized by the transcription factor AP-1 [49]. The binding of AP-1 to TRE could also initiate MCP-1 transcription [50]. As we know, AP-1 can be activated by the upstream three functional parallel MAPKs, including JNK, p38 and ERK [51], and inhibiting whichever kinase will suppress the AP-1 signalling. To address the causal relationship between AP-1 signalling and MCP-1 production, the inhibitors of JNK, p38 and ERK were used. Unlike NF-κB signalling, inhibiting the phosphorylation of MAPKs significantly reduced MCP-1 production (Figure 5), demonstrating that MAPKs/AP-1 pathway plays a key role in MCP-1 production in TNF-α-stimulated adipocytes. Perhaps not coincidentally, the overexpression of c-jun and c-fos, the members of AP-1 complex, markedly elevated MCP-1 gene expression through NF-κB-independent mechanism in HUVECs [52].

Generally, NF-κB-like and AP-1 consensus binding sites are required for MCP-1 gene expression induced by cytokines, including TNF-α, in multiple cells [53,54]. Indeed, the constitutive activation of NF-κB may play important roles in the regulation of many inflammatory mediators. Nevertheless, our study revealed, for the first time, that a series of NF-κB inhibitors with different mechanisms failed to inhibit MCP-1 production induced by TNF-α in adipocytes. Instead, MAPKs/AP-1 signalling is predominant. In view of the important role of MCP-1 in adipose tissue inflammation, our findings provide important insight into the outstanding role of AP-1 signalling in adipose tissue, and may lead to the development of therapeutical repositioning strategies in metaflammation.

## Materials & methods

### Reagents

DMEM (Cat No. 1,927,569) was purchased from Gibco BRL. FCS (Cat No. 22,011–8612) was obtained from Zhejiang Tianhang Biotechnology Co. Recombinant mouse TNF-α protein (Cat No. 50,349-MNAE) was obtained from Sino Biological Inc. INH14 (Cat No. HY-114454) and cycloheximide (CHX, Cat No. HY-12320) were purchased from MedChemExpress. Dexamethasone (Cat No. D3628), BAY11-7082 (Cat No. T2846), APDC (Cat No. P0644), TPCK (Cat No. T2810) were purchased from Tokyo Chemical Industry. JSH-23 (Cat No. 481,408) was obtained from Sigma-Aldrich. B022 (Cat No. GC39280), calpeptin (Cat No. GC1034), AS601245 (Cat No. GC10010) and SCH772984 (Cat No. GC16001) were from GlpBio Technology Co. Collagenase (Cat No. S10053) and rosiglitazone (Cat No. B21439) were from Shanghai Yuanye Bio-Technology Co. Mouse MCP-1 ELISA kit (Cat No. 432,701) was produced by BioLegend Co. TRIzol Reagent (Cat No. 15596026) was from Invitrogen Co., and M-MuLV first strand cDNA Synthesis kit was from Sangon Biotech Co. PD169316 (Cat No. SD5946), SP600125 (Cat No. S1816), FR180204 (Cat No. SD5978), SB203580 (Cat No. S1863), insulin (Cat No. P3375), NFκB-TA-luc (Cat No. D2207), AP1-TA-luc (Cat No. D2108) reporter plasmids and luciferase assay system (Cat No. RG005) were purchased from Beyotime Institute of Biotechnology. Entranstera™-H4000 (Cat No. 4000–5) was purchased from Engreen Biotechnology Co. All other reagents were of analytical grade.

### Cells

3T3-L1 pre-adipocytes were obtained from Cell Center of Institute of Basic Medical Sciences, Chinese Academy of Medical Sciences (Beijing, China). They were cultured in DMEM containing 10% heat-inactivated FCS in a humidified incubator with 5.0% CO_2_ at 37°C.

3T3-L1 pre-adipocytes were differentiated into mature adipocytes as previously described with modifications [55]. Briefly, 3T3-L1 pre-adipocytes were cultured in basal medium (DMEM containing 10% heat-inactivated FCS) until confluence. After about 2 days, cell differentiation was induced by changing the medium to the M1 medium (basal medium with the addition of 1.5 μg/mL insulin, 1 μM dexamethasone, 0.5 mM IBMX and 2 μM rosiglitazone). Two days later, the medium was changed to M2 medium (basal medium with 1.5 μg/mL insulin). Two days later, the medium was replaced with M1 medium for another 2 days. Cells were differentiated and maturated when a lot of lipid droplets could be observed by the microscope.

Mouse primary adipocytes were isolated from male C57BL/6 N mice as described previously with modifications [56]. In brief, mice were anesthetized by an intraperitoneal injection of 2,2,2-tribromoethanol (200 mg/kg) and sacrificed via a dislocated neck. The epididymal fat pads were removed by excision and digested by collagenase (2 mg/mL in DMEM) with BSA (20 mg/mL) at 37°C for 1 h. Mouse primary adipocytes were obtained through being sifted by a fine-mesh sieve (0.25 mm) and cultured in DMEM containing 10% heat-inactivated FCS in a humidified incubator with 5.0% CO_2_ at 37°C.

### Animals

The male C57BL/6 N mice (18 g – 20 g) were from Vital River Experimental Animal Services (Beijing, China) (licence number: SYXK (Beijing) 2017–0020) and housed in an SPF laboratory. All experiments were approved by the Institutional Care and Use Committee of the Institute of Medicinal Plant Development (IMPLAD) of Chinese Academy of Medical Sciences and carried out according to the Guidelines for the Care and Use of Laboratory Animals (8th edition). Anaesthetic drugs and all other necessary measures were used for alleviating animal suffering during the experimental procedures.

### Measurement of MCP-1 level

The concentration of MCP-1 was measured using commercial ELISA kit. Adipocytes (4 × 10^5^ cells per well in 96-well plates) were pretreated with multiple inhibitors for 1 h and followed by mouse TNF-α stimulation at 37°C for 24 h. Supernatant or subnatant MCP-1 was assayed using ELISA kit according to the manufacturer’s instruction. The concentration of MCP-1 was calculated from the standard curve.

### Transfections and luciferase assays

3T3-L1 pre-adipocytes were transfected with plasmid including pNFκB-TA-luc or pAP1-TA-luc by Entranstera™-H4000 according to the manufacture’s instruction. The transfected cells (1 × 10^6^ cells per well in 24-well plates) were stimulated by mouse TNF-α (20 ng/mL) for 4 h. The cells were lysed, then the luciferase activity in the lysate was measured using the luciferase assay system according to the manufacturer’s instruction.

### RNA extraction and quantitative real-time PCR (RT-qPCR)

Total mRNA was isolated from adipocytes by Trizol reagent according to the manufacturer’s instruction. Reverse transcription reactions were conducted according to the manufacturer’s instruction of the M-MuLV first strand cDNA synthesis kit. The RT-qPCR analyses were performed on a BIOER Fluorescent Quantitative Detection System (Bioer Technology, Hangzhou, China). The procedure condition was as follows: 95°C for 20s, followed by 40 cycles of denaturation at 95°C for 15s and annealing/extension at 60°C for 20s. The comparative Ct method (2^−ΔΔCt^) was used to analyse the relative intensities of the inflammatory genes. The MCP-1 primer sequences were detailed as follows: 5’-GCC CCA CTC ACC TGC TGC TAC T-3’ (forward) and 5’-CCT GCT GCT GGT GAT CCT CTT GT-3’ (reverse). For β-actin, used as a control, the primes were: 5’- TGT TAC CAA CTG GGA CGA CA-3’ (forward) and 5’- AAG GAA GGC TGG AAA AGA GC-3’ (reverse).

### Statistical analysis

All statistical analyses were performed with the GraphPad Prism (version 7.0). Comparisons between two groups were performed using an unpaired Student’s *t*-test. Comparisons between multiple treatment groups were performed using one-way ANOVA with the Tukey’s *post hoc* analysis. All data were reported as mean ± SD of at least three independent experiments. *P* < 0.05 was considered statistically significant.

## Data Availability

The data that support the findings of this study are openly available in [Medeleley Data] at https://data.mendeley.com/drafts/3frm3mnkpc, reference number [DOI: 10.17632/3frm3mnkpc.1].
